# Effect of the Chinese Medicine YangZheng XiaoJi on Reducing Fatigue in Mice with Orthotopic Transplantation of Colon Cancer

**DOI:** 10.1155/2019/3870812

**Published:** 2019-02-12

**Authors:** Yanni Zhang, Zhongxin Li, Zhaolong Zhao, Wentao Kuai, Cong Wei, Jian Lv, Jie Zhi, Yitao Jia

**Affiliations:** ^1^Second Department of Surgery, the Forth Hospital of Hebei Medical University, 169 Tianshan Road, Shijiazhuang, Hebei 050000, China; ^2^Department of General Surgery, Shanghai Ninth People's Hospital, Shanghai Jiao Tong University School of Medicine, 639 Zhi Zao Ju Road, Shanghai 200011, China; ^3^Department of Oncology, Hebei General Hospital, 348 Heping West Road, Shijiazhuang, Hebei 050051, China; ^4^National Key Laboratory of Collateral Disease Research and Innovative Chinese Medicine, Yiling Medical Research Institute, 238 Tianshan Road, Shijiazhuang, Hebei 050035, China

## Abstract

**Background:**

Fatigue is a common, distressing, and persistent symptom for patients with malignant tumor including colorectal cancer (CRC). Although studies of cancer-related fatigue (CRF) have sprung out in recent years, the pathophysiological mechanisms that induce CRF remain unclear, and effective therapeutic interventions have yet to be established.

**Methods:**

To investigate the effect of the traditional Chinese medicine YangZheng XiaoJi (YZXJ) on CRF, we constructed orthotopic colon cancer mice, randomly divided into YZXJ group and control (NS) group. Physical or mental fatigue was respectively assessed by swimming exhaustion time or suspension tail resting time. At the end of the experiment, serum was collected to measure the expression level of inflammatory factors by ELISA and feces to microbiota changes by 16s rDNA, and hepatic glycogen content was detected via the anthrone method.

**Result:**

The nutritional status of the YZXJ group was better than that of the control group, and there was no statistical difference in tumor weight. The swimming exhaustion times of YZXJ group and control group were (162.80 ± 14.67) s and (117.60 ± 13.42,* P* < 0.05) s, respectively; the suspension tail resting time of YZXJ group was shorter than that of the control group (49.85 ± 4.56) s and (68.83 ± 7.26) s,* P* < 0.05)). Serum levels of IL-1*β* and IL-6 in YZXJ group were significantly lower than the control group (*P* < 0.05). Liver glycogen in YZXJ group was (5.18 ± 3.11) mg/g liver tissue, which was significantly higher than that in control group (2.95 ± 2.06) mg/g liver tissue (*P* < 0.05). At phylum level, increased abundance of* Bacteroidetes, Verrucomicrobia, Actinobacteria, *and* Cyanobacteria* and decreased* Proteobacteria* in YZXJ group emerged as the top differences between the two groups, and the* Firmicutes/Bacteroidetes* ratio was decreased in YZXJ group compared to the control group. At genus level, the abundance of* Parabacteroides*, unidentified* Saprospiraceae, *and* Elizabethkingia* which all belong to phylum* Bacteroidetes* were increased, while* Arcobacter, Marinobacter, Alkanindiges, Sulfuricurvum, Haliangium*, and* Thiobacillus* in phylum* Proteobacteria* were decreased after YZXJ intervention. YZXJ can also increase* Pirellula*,* Microbacterium,* and* Alpinimonas* and decrease* Rubrobacter* and* Iamia*.

**Conclusion:**

YZXJ may reduce the physical and mental fatigue caused by colorectal cancer by inhibiting inflammatory reaction, promoting hepatic glycogen synthesis, and changing the composition of intestinal microbiota.

## 1. Introduction

Cancer-related fatigue (CRF) is one of the common, suffering and persistent symptoms of cancer patients, often accompanied by the entire course of malignancy. The prevalence of CRF may vary with an estimate of 60%–96% in malignant tumor patients who are undergoing treatment and experiencing fatigue [1, 2]. Unlike the transient fatigue of normal healthy people, the incidence of CRF is sudden, with the subjective sense of tiredness or exhaustion which is not proportional to recent physical activity and interferes with usual functioning; rest or sleep cannot alleviate it [3]. Due to the diversity of clinical manifestations of malignant tumors, CRF often appears as part of a symptom cluster (including cachexia and neuropsychological disorders) [4]. CRF, including physical and mental fatigue, directly or indirectly affects the physical and mental state of cancer patients, reducing quality of life and compliance with treatment, which is an important issue that cannot be ignored in the treatment of cancer.

Although the exact mechanisms have not yet been clearly elucidated, what can be confirmed is that multiple factors interact to cause the incidence of CRF, both central and peripheral factors. The former includes cytokine dysregulation, hypothalamic-pituitary-adrenal (HPA) axis disruption, circadian rhythm, 5-hydroxytryptamine (5-HT), vagal afferent nerve function, while peripheral factors mainly refer to energy metabolism abnormalities, such as adenosine triphosphate (ATP) deficiency and muscle acidosis [5–7]. In colorectal cancer (CRC) patients, the severity of CRF is closely related to nutrition status, especially white blood cells and serum calcium levels [8]. A growing body of evidence suggests that the tumor itself, the patient's mental stress, or chemoradiotherapy mediates a low-grade inflammatory response that is inextricably linked to the development of CRF. Recent studies also indicate that interleukin-1 (IL-1), interleukin-6 (IL-6), soluble tumor necrosis factor receptor type II (sTNF type II), and C-reactive protein (CRP) have played a significant contribution [9]. These inflammatory factors can both act directly on the central nervous system and affect the HPA axis [10, 11]. Some scholars also have found that elevated levels of IL-1*β* in peripheral blood activate the vagus nerve, which in turn leads to fatigue [12].

Metabolic abnormalities play an important role in peripheral causal factors, including immune dysregulation, mitochondrial dysfunction, 5′-adenosine monophosphate-activated protein kinase activation, and skeletal muscle cell acidosis, which may cause different degrees of insufficient energy synthesis [13, 14]. Hepatic glycogen is a form of energy storage in the body. During vigorous exercise or pathological conditions, energy consumption increases, and hepatic glycogen is decomposed into glucose, which provides energy for muscles and helps to maintain endurance. Data showed that liver glycogen in fatigue patients was generally reduced, while appropriate supplementation could alleviate fatigue [15]. Chronic inflammation, as well as chemoradiotherapy and many other factors, can cause metabolic abnormalities. For example, chronic inflammation induces insulin resistance, as a consequence of a decrease in carbohydrate synthesis glycogen and also increases energy expenditure by about 10% [16, 17]. Yet it is true that mitochondria are usually inevitably damaged in routine clinical practice, which also causes insufficient energy supply [18].

In recent years, the relationship between intestinal microbiota imbalance and host health has attracted more attention, including fatigue syndrome, suggesting that the brain-gut axis may play an important role in the occurrence of mental fatigue. Researchers have found that in the intestines of patients with chronic fatigue syndrome (CFS), the abundances of* Alistipes, Lactonifactor, Streptococcus spp., Enterococcus spp.*,* Prevotella spp.*, etc., are increased; however, some probiotics such as* Bifidobacteria* are reduced [19, 20]. It is particularly noteworthy that intestinal microecological disorders are closely related to the occurrence and development of CRC. Due to the special positional relationship, the intestinal microbiota is actually an important part of the CRC microenvironment. Therefore, CRF in patients with CRC may have a special mechanism different from that of other solid tumors; that is, in addition to inflammatory reactions and metabolic abnormalities, there may exist such mechanism as intestinal microbiota imbalance. Increased abundances of* Streptococcus gallolyticus, Enterococcus faecalis, Bacteroides fragilis, Prevotella, Helicobacter, and Fusobacterium nucleatum* and decreased probiotics like* Lactobacilli* and* Bifidobacteria* are detected in CRC patients by previous reporting [21, 22]. Likewise, fatigue is considered to have an association with increased bacterial growth as* Enterococcus and Prevotella* or decreased abundance of probiotics [23, 24].

Such a complex pathogenesis of CRF means that the treatment cannot be unique or single. Although guidelines have been issued, lots of clinical obstacles need to be overcome. Current treatment strategies for CRF include nondrug and drug therapy. Nondrug therapies contain physical exercise, cognitive behavioral therapy, and psychosocial intervention [25]. The study suggested that aerobic combination exercise was the first recommendation rather than simple resistance training; moreover, light to moderate physical activity is beneficial, and otherwise strenuous activities can be counterproductive [26]. For drug therapy, a meta-analysis showed that there is no specific drug for the treatment of CRF, and some studies have shown a preference for modafinil and methylphenidate. As for Erythropoietin, Dexamethasone, acetylsalicylic acid, methylprednisolone, armodafinil, amantadine, and L-carnitine, the efficacy remains to be further observed. Meanwhile, toxic side effects of these preparations also limit the widespread and long-term clinical use [27, 28].

Thus, seeking an integrative treatment program is the future direction [29]. Chinese traditional medicine has its unique advantages in this respect. For example, except for the well-known Ginseng, some Chinese herbal formulas also have good antifatigue effects [30–33]. Acupuncture is considered a beneficial alternative treatment to CRF in patients with breast cancer or undergoing anticancer therapy [34]. However, it should be noticed that most of these methods do not treat tumors, and even some of them may be contraindicated. Therefore, it is necessary to develop a new prescription to treat CRF. YangZheng XiaoJi (YZXJ) is recently developed, consisting of 16 traditional Chinese medicines named Astragalus membranaceus, Fructus ligustri lucidi, Ginseng, Rhizoma curcumae, Ganoderma, Gynostemma pentaphyllum, Atractylodes macrocephala, Scutellaria barbata, Hedyotis diffusa, Poria cocos, Eupolyphaga seu steleophaga, Endothelium corneum gigeriae galli, Mock strawberry herb, Bittersweet herb, Herba artemisiae scopariae, and Cynanchum paniculatum. Chromatographic analysis showed that the main components were oleanolic acid and ursolic acid. Jiang et al. found that YZXJ inhibits tumor growth by antagonizing HGF/c-Met and inhibiting angiogenesis [35, 36]. At present, a large randomized controlled clinical trial of YZXJ for CRF was conducted in several Chinese hospitals (Registration number: NCT02195453). The primary end point of the trial was the effect of YZXJ on the fatigue of patients with advanced lung cancer, which results will be announced soon and are worth looking forward to. The mechanism of YZXJ anti-CRF also needs to be clarified.

In this study, we established the orthotopic implantation model of CT26 cells in Balb/c mouse to explore the efficacy of YZXJ in the treatment of CRF caused by CRC, and the related mechanism, aiming to provide experimental basis for finding a new method for treating CRF in patients with CRC.

## 2. Materials and Methods

### 2.1. Cancer Cell Line

CT26 cell line used for these experiments was purchased from the Shanghai Cell Collection Committee, maintained in tissue culture flasks (5% CO_2_, air at 37°C) containing RPMI-1640 (Invitrogen) with 10% newborn calf serum (Invitrogen). Trypsinization and inheritance were carried out with EDTA/pancreatin mixture (Gibco). When reaching the logarithmic growth phase, CT26 cells were prepared as single cell suspension at a concentration of 2×10^7^/ml.

### 2.2. Orthotopic Mouse Model

Forty-two SPF raised 6- to 7-week-old BALB/c mice (male) were obtained from the Animal Center of Hebei Medical University, weighing about 18~22 g, raised in the SPF-level laboratory of the Animal Experimental Center of the Fourth Hospital of Hebei Medical University, room temperature 25°C ± 1°C. All operations were carried out according to the Ethics Committee of Hebei Medical University.

After a 7d acclimatization period, tumors were started by injecting CT26 cell suspension at a dose of 0.1ml/mouse to the right flank of two mice. Two weeks later, the subcutaneous tumors which grown to a diameter of about 1 cm were isolated and cut into 2 mm × 2 mm × 1 mm size and reserved in normal saline. The other mice were also anesthetized with 3% pentobarbital sodium 40~45 mg/kg, routinely disinfected the skin then opened the abdominal cavity, gently scraped off the serosal surface on the opposite side of mesentery at the colon of about 1cm from the ileocecal junction. The prepared fresh tumor mass was placed in the formed “diverticulum” with squeezing a medical drop to seal the tumor nest and close the abdomen after returning cecum.

### 2.3. Experimental Design

Mice were randomized to experimental (YZXJ) group and control (NS) group (n = 40). On the 2nd day after building model, the experimental group was administered with YZXJ suspension (Yiling pharmaceutical, Shijiazhuang, China) at a dose of 1.6 g/kg/d for 21 days, and the control group was given a corresponding volume of normal saline. Mice were sacrificed under anesthesia with removing and weighing the abdominal colorectal tumors on the 21st day.

#### 2.3.1. Observation of the Fatigue State

*(1) Recording the General Condition*. Changes in hair appearance, mental state, activity, food intake (weighed every 4d), and body weight (every 1w) of the mice in each group were observed and recorded.

*(2) Determination of Physical Fatigue*. Physical exhaustion was used to assess somatic fatigue. Three days before modeling, all mice were placed in a swimming box (water temperature 25°C ± 1°C) for adaptive training, 5 min/d. On the 10th day after administration, exhaustive swimming time was recorded by dropping 7% body weight of lead on the tail, which standard is the nose tip sinking into the water for 6 s.

*(3) Determination of Mental Fatigue*. On the 14th day, we fixed the tail on a tail suspension tester with a tape in a dark room and kept mouse head 5 cm away from the bottom of tester. After 2 min of adaptive suspension, the mice resting times were recorded in the last 4 min.

#### 2.3.2. Detection of Inflammatory Factors in Serum

Blood samples were taken from eyeball at the end of the experiment. After standing for 30 min, it was centrifuged at 3000 r/min for 10 min, and the supernatant was stored in a -80°C refrigerator until further analysis for detecting cytokine levels of plasma IL-1*α*/*β*, IL-2, IL-6, IL-10, TNF-*α*, IFN-*γ* according to ELISA kit (MultiSciences).

#### 2.3.3. Comparison of Hepatic Glycogen

The liver tissue was weighed 100 mg, boiling with 300 *μ*l concentrated alkaline for 20 min to destroy other components except liver glycogen. Then, we mixed 1% hepatic glycogen detection solution prepared by double distilled water and 2 ml anthrone developer dissolved in 95% concentrated sulfuric acid (Nanjing Jiancheng Bioengineering Institute) and continued boiling for 5 min; the absorbance of each measuring tube and standard glycogen detection solution (0.001 mg) was measured at a wavelength of 620 nm after natural cooling, and zero was adjusted with a blank tube. The content of hepatic glycogen was calculated by the following formula.

(1) Hepatic  glycogen  mg/g  tissues=Measuring  tube  OD  valueStandard  tube  OD  value×Measuring  tube  content  0.01mg×Sample  dilution  ratio×10÷1.11

#### 2.3.4. Determination of Intestinal Microbiota

After administering for 21 days, the feces (about 1 g/mouse) were collected using a sterile eppendorf tube and frozen at -80°C. The effect of YZXJ on the intestinal microbiota of orthotopic colon cancer mice was detected by 16S rDNA; a small fragment library was constructed for single-end sequencing based on the IonS5TM XL platform, which revels the species composition of samples through the cut filtering of reads, OTUs (Operational Taxonomic Units) clustering, species annotation and abundance analysis.

### 2.4. Statistical Analyses

Data were expressed as mean ± SD analyzed by t test, and those who did not meet the positive distribution were tested by rank sum test. All the Clean Reads of samples were clustered by Uparse software, and the sequence was clustered into OTUs with 97% identity. Meanwhile, the sequence with the highest frequency in OTUs was used as the representative sequence. Through specimen annotation of OTUs representative sequences using the Mothur method and SSUrRNA database (set threshold value as 0.8~1), taxonomic information can be obtained to calculate out community composition of each sample in kingdom, phylum, and class, order, family, genus, respectively. Fast multisequence alignment was performed using MUSCLE software (Version 3.8.31) to obtain a systematic relationship of all OTUs representative sequences. The Qiime software (Version 1.9.1) was used to calculate the Chao1, Simpson index and Unifrac distance, and the R software (Version 2.15.3) was used to analyze the difference between *α* and *β* diversity index groups. Statistical analysis was performed using SPSS (Version 21.0). All results were considered statistically significant at* P* < 0.05.

## 3. Results

**Figure 1:**
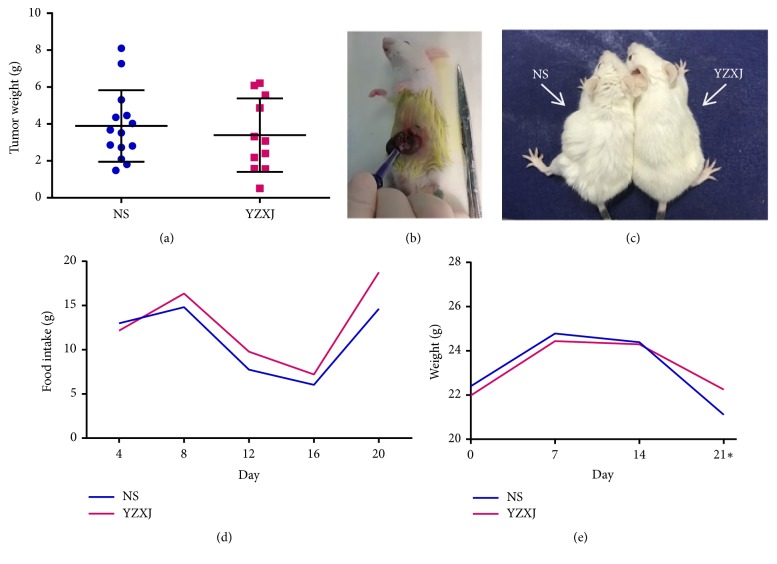
General condition of the two groups. (a) Tumor weight comparison of NS and YZXJ groups. No statistically significant difference was observed between the two groups. (b) Colon cancer orthotopic transplantation in BALB/c mice. (c) Appearance and activity. NS group had rougher, lusterless hair, more apathetic condition, and lower movement than YZXJ group. (d) Average daily food intake of each mouse weighted every 4 days. (e) Average weight of each mouse weighted every week. *∗* Weight without in situ tumor.

Totally 40 mice underwent surgical operation. Finally, 14 of NS group and 11 of YZXJ group were successfully modeled. In the end, the orthotopic tumor mass of NS group was (3.90 ± 0.52) g, and YZXJ group was (3.40 ± 0.60) g, which was not statistically significant (t = 0.63,* P* = 0.53, Figures 1(a) and 1(b)).

### 3.1. Impact on the General Condition

#### 3.1.1. Appearance

Before administration, there were no differences between the two groups in mental state and activity. During experiment, mice in both groups gradually showed different degrees of poor gloss in hair, slowness of movement, and reduced food intake. Until the end of observation, mice in NS group had rougher hair and less lusters than YZXJ group, as well as apathetic and slow action, while mice in YZXJ group appeared more flexible (Figure 1(c)).

#### 3.1.2. Food Intake

The average food intake of mice was evaluated every 4 days. As depicted in Figure 1(d), YZXJ group had more food intake than NS group and also presented an increasing food intake with the prolonged administration time, but the difference did not show statistical significance.

#### 3.1.3. Body Weight

Body weight was measured every week, and tumor weight was subtracted from it in the last time. Although the difference in body weight between the two groups did not reach statistical significance, by the end of experiment, the weight loss trend of YZXJ group was slower than that of NS group (Figure 1(e)).

### 3.2. Impact on Fatigue

#### 3.2.1. Physical Fatigue

**Figure 2:**
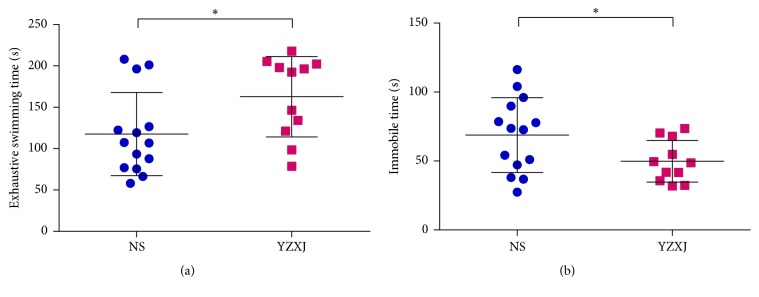
Comparison of fatigue state. (a) Physical fatigue exhaustive swimming time of NS and YZXJ group tested after 10-day intervention. (b) Immobile time in tail suspension test of NS and YZXJ group tested after 14-day intervention. *∗ P*<0.05.

Exhaustive swimming time is shown in Figure 2(a). The swimming time of each mouse was close before modeling, but on the 10th day after intervention, exhaustive swimming time of YZXJ group was (162.80 ± 14.67) s, longer than that of NS group (117.60 ± 13.42) s, (t = 2.27,* P* = 0.03).

#### 3.2.2. Mental Fatigue

Figure 2(b) is the result of suspension tail experiment. It can be seen that the tail suspension resting time in YZXJ group was shorter than that in NS group [(49.85 ± 4.56) s vs. (68.83 ± 7.26) s, t = 2.22,* P* = 0.04]. This suggested that the energy and mental state of mice with orthotopic colon cancer were better in those taking YZXJ.

### 3.3. Impact on Serum Inflammatory Factors

**Figure 3:**
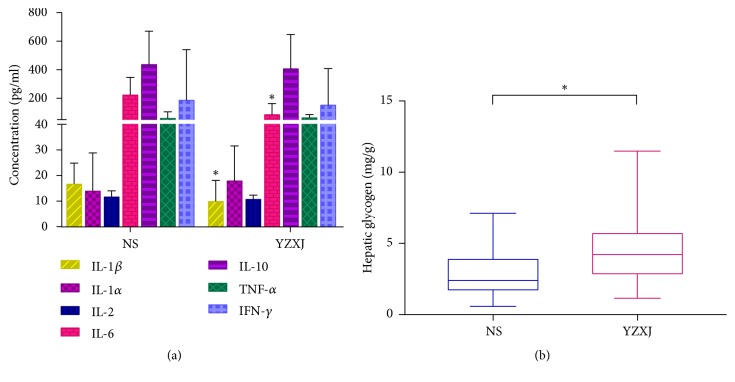
Concentration of inflammatory factors and hepatic glycogen. (a) Fatigue-related inflammatory factors in YZXJ group were generally lower than NS group, particularly for IL-1*β* and IL-6. (b) The level of liver glycogen in the two groups, reflexing the advantage energy reserve capacity in YZXJ treated mice. *∗ P*<0.05.

To determine the possible factors of improving the fatigue performance, inflammatory factors levels associated with fatigue which once have been reported were tested in mice serum, including IL-1*α*/*β*, IL-2, IL-6, IL-10, TNF-*α*, IFN-*γ*. In expectation, fatigue-related inflammatory factors in YZXJ group were generally lower than NS group, particularly IL-1*β* and IL-6. The level of IL-1*β* in YZXJ group was significantly lower than NS group [(10.29 ± 7.86) pg/ml vs. (17.07 ± 7.84) pg/ml,* P* = 0.04]. The IL-6 level in YZXJ group was (92.40 ± 71.43) pg/ml, and NS group was (229.44 ± 117.54) pg/ml (*P* = 0.002) (Figure 3(a), Table 1).

### 3.4. Determination of Hepatic Glycogen

To investigate the effect of YZXJ on hepatic glucose metabolism, we detected liver glycogen. The results showed that the level of hepatic glycogen in NS group was (2.95 ± 2.06) mg/g, which increased to (5.18 ± 3.11) mg/g in YZXJ group (*P* = 0.04, Figure 3(b)), indicating YZXJ treatment was advantageous to promote reserve capacity of tumor-bearing mice.

### 3.5. Changes in Intestinal Microbiota

**Figure 4:**
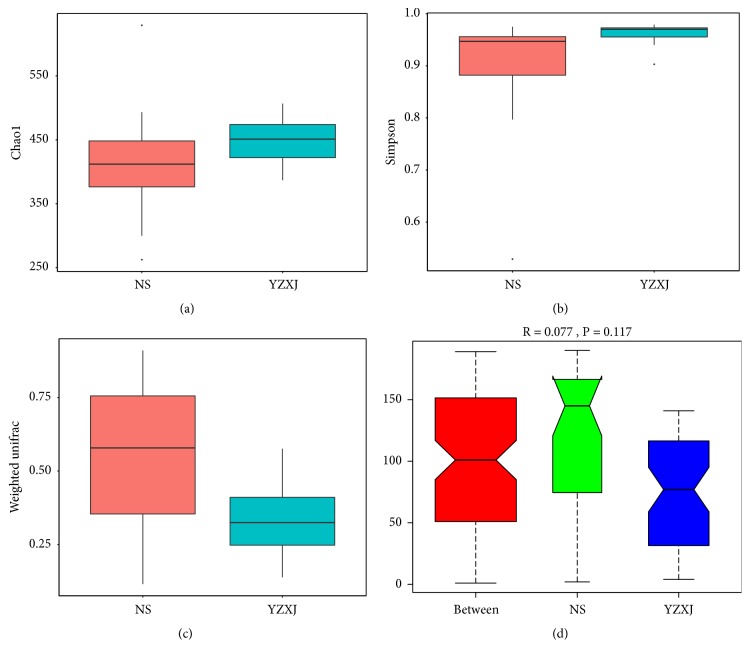
Comparison of *α* and *β* diversity. (a, b) Two indicators of *α* diversity detected in NS and YZXJ group. Chao 1 is represented as the bacteria abundance, and Simpson index reflects the bacteria diversity. (c) Unifrac distance of *β* diversity is used to calculate the distance between samples by using the evolutionary information and further constructing weighted Unifrac distance through OTUs abundance information. (d) Intergroups difference was greater than intragroup difference, but the difference was not statistically significant (*P*=0.117).

To explore the influence of YZXJ on intestinal microbiota, 16s rDNA was used to detect the changes of intestinal microbiota in mice. The result displayed that comparison of *α* diversity including Chao 1 and Simpson index of YZXJ group were higher than NS group, which represent the bacteria abundance and diversity, respectively (Figures 4(a) and 4(b)). However comparison in *β* diversity indicated that microbial community composition of YZXJ group was lower than NS group. ANOSIM analysis showed that the community structure difference between groups was greater than that within the group, but the difference did not reach statistical significance (*P* = 0.117, Figures 4(c) and 4(d)).

**Figure 5:**
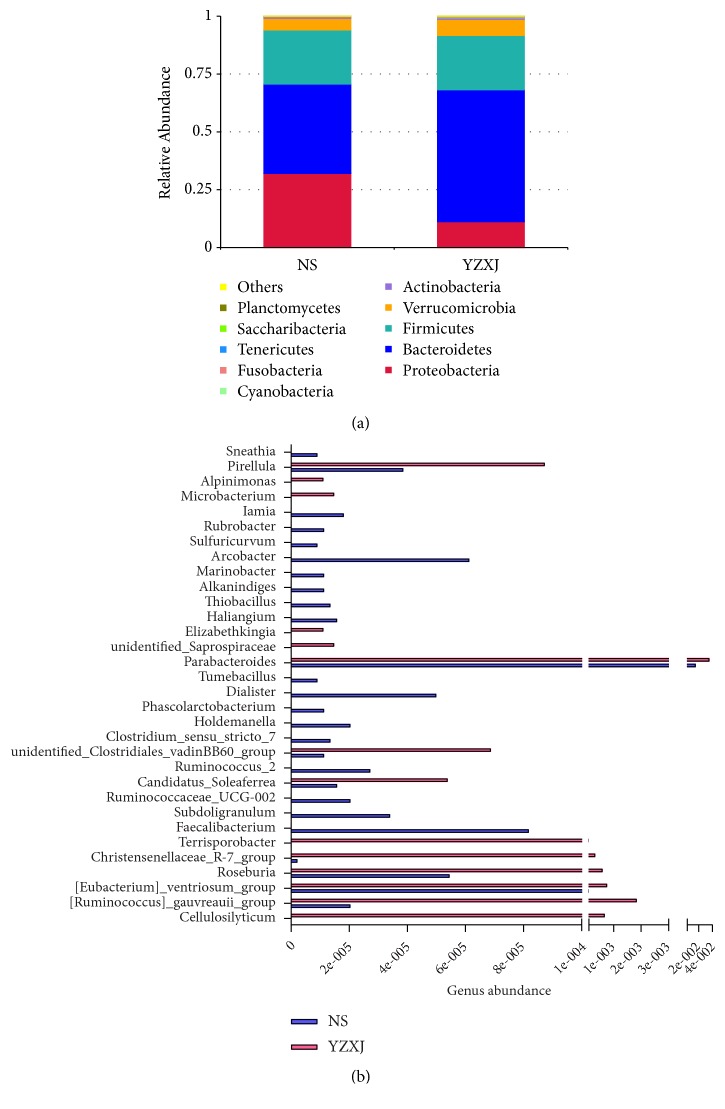
Alteration of fecal microbiota due to the intervention of YZXJ. (a) Cumulative column chart of relative species abundance in phylum level. (b) Top differences of fecal microbiota shifts in genus level.

In order to study the species with significant differences between groups, MetaStat method was used to test the species abundance at different levels. Results showed that at phylum level, increased abundance of* Bacteroidetes, Verrucomicrobia, Actinobacteria*, and *Cyanobacteria* and decreased* Proteobacteria* in YZXJ group emerged as the top differences between two groups (Figure 5(a)). The* Firmicutes/Bacteroidetes* ratios were 0.61 and 0.41 in NS and YZXJ group, respectively. At genus level, the abundance of* Parabacteroides*, unidentified* Saprospiraceae*, and* Elizabethkingia* which all belong to phylum* Bacteroidetes* were increased, while* Arcobacter, Marinobacter, Alkanindiges*,* Sulfuricurvum, Haliangium, Thiobacillus* in phylum* Proteobacteria* were decreased after YZXJ intervention, which also increased* Pirellula*,* Microbacterium,* and* Alpinimonas* and decreased* Rubrobacter* and* Iamia* (Figure 5(b), Table 2).

## 4. Discussion

CRF, a serious concomitant symptom of most cancer patients, is one of the problems that plague clinicians for its indefinite therapeutic strategy due to the ambiguous pathogenesis. In this study, we found that the traditional Chinese medicine prescription of YZXJ can significantly alleviate physical and mental fatigue of mice with orthotopic colon cancer and not promote tumor growth. Further studies revealed that YZXJ can reduce the serum inflammatory factors IL-1*β* and IL-6, increase hepatic glycogen content, affect the composition of intestinal microbiota, mainly increase the abundance of* Bacteroides*, and also decrease the abundance of* Proteobacteria* and the* Firmicutes/Bacteroidetes* ratio. This study aimed to provide a basis for clinical search for a new method of CRF treatment and partially elucidate the mechanism of Chinese medicine in treating CRF.

At present, the most accepted view on the pathogenesis of CRF is inflammatory response. We found that YZXJ can reduce various inflammatory factors in mice blood, with the most significant decrease in IL-1*β* and IL-6. It is well known that tumor cells or mesenchymal cells in surrounding microenvironment can release a variety of inflammatory factors, so will the conventional anticancer treatments such as radiotherapy and chemotherapy. Such chronic inflammation can induce fatigue syndrome through the following mechanisms: (1) triggering metabolic disorders, as a result of insufficient energy supply to cells; (2) increasing energy consumption of immune system, resulting in impaired immune function; and (3) causing neuroendocrine dysfunction, neurotransmitter metabolism disorder, or abnormal neuronal formation [37, 38]. Studies have shown that anti-inflammatory treatment can relieve fatigue; for example, selective 5-HT reuptake inhibitors exhibit the most beneficial effects in restraining the inflammation markers in patients with depression [39]. Certain anti-inflammatory preparations can also attenuate fatigue symptoms [40, 41]. However, in the process of theoretical research towards clinical application, there still exist many problems waiting to be solved, such as safety, and there are no commercially available preparations to choose from as well.

YZXJ contains ingredients with heat-clearing and detoxifying properties such as Scutellaria barbata, Bittersweet herb, and Herba artemisiae scopariae. Scutellaria barbata, a traditional anticancer Chinese medicine, has been found to have the additional effect of anti-inflammation and anti-infection in recent years. Diterpenoid alkaloids and 6-methoxynaringenin in the Scutellaria barbata extracts had the strongest effect on downregulating the secretion of NO, PE2, IL-6, IL-1*β* induced by LPS and P-JNK signaling pathway activation [42–44]. Moreover, the water extracts of Bittersweet herb and Herba artemisiae scopariae can alleviate inflammation by inhibition of NF-*κ*B pathway that reduces the generation of proinflammatory cytokines like IL-1*β* and IL-6 [45–47]. Other components in YAXJ such as Astragalus membranaceus have the effects of invigorating Qi, generating Yang, strengthening exterior, and stopping sweating, often used for the rehabilitation of patients with weak constitution. However, recent studies have found that the extract Astragalus polysaccharide can inhibit the production of inflammatory factors such as TNF-*α* or IL-1*β* through NF-*κ*B, ERK, JNK, and other pathways to regulate immune function and reduce intestinal inflammation [48, 49]. In addition, Ganoderma lucidum polysaccharide extracted from Ganoderma, one of the components of YZXJ, have beneficial effects on improving CRF in breast cancer patients without any significant adverse effect [50]. Further animal experiments suggested that, in addition to slowing down the growth of tumors, Ganoderma lucidum polysaccharides can also prolong exhausting swimming time of tumor-bearing mice through decreasing the level of TNF-a or IL-6 induced by cisplatin and upregulating the SOD activity in the muscle [51].

Except for the inflammation, oxidative stress, energy metabolism, and abnormal hormone levels are also important mechanisms for the development of CRF, especially physical fatigue. Emerging evidence demonstrated that persistent fatigue occurs when the body kinetic energy consumption exceeds the cells energy supply capacity. Physical fatigue is almost accompanied by metabolic disorders, mainly liver glycogen and muscle glycogen metabolism [52]. In fact, behind the metabolic disorders we can still see the “shadow” of inflammatory responses, which affect the host metabolism and neuroendocrine reactions and induce or promote fatigue symptoms [53]. The tumor itself, as an inflammatory response, often appears simultaneously with a reduction in hepatic glycogen [54, 55]. Therefore, we examined the effect of YZXJ on hepatic glycogen levels. As expected, YZXJ can increase the reserve of liver glycogen, which is consistent with the literature. A number of studies have confirmed that increasing glycogen levels may alleviate fatigue; for example, hydrogen water drinking relieves fatigue by lowering blood sugar, lactic acid, BUN, and increasing liver glycogen [56]. In the mouse swimming experiment, acute valine supplementation helps maintain liver glycogen and blood sugar levels and increases spontaneous activity, which could contribute to relieving postexercise fatigue [57]. For a long time, people have tried to adopt traditional Chinese medicine methods such as Ginseng and fungus to treat fatigue and achieved good results. Basic research found that most of these methods increase glycogen content in liver and muscles [58–60]. In addition to ingredients that add supplemental energy such as Ginseng and ingredients like Eupolyphaga seu steleophaga that reduce tissue oxygen consumption, YZXJ relieves fatigue from both entry and exit.

Flora imbalance is involved in the development of CRC by affecting inflammatory response or immune status; on the other hand, it can induce fatigue through the inflammatory or metabolic pathways. Among the numerous bacteria associated with CRC,* Fusobacterium, Bacteroides fragilis (Prevotella), Shigella, Helicobacter*, etc. can promote inflammation, conversely,* Clostridium*, especially* Faecalibacterium prausnitzii*, can inhibit the inflammatory response. Meanwhile, the proinflammatory intestinal flora mentioned above can cause fatigue, whereas* Clostridium, Bifidobacterium*, and* Faecalibacterium* can alleviate fatigue. In view of these above, people are trying to treat CRF from the perspective of regulating intestinal microbiota.

In order to study the effect of YZXJ on intestinal microbiota, we used a method of colon cancer orthotopic transplantation in BALB/c mice to restore the growth environment of colon cancer as much as possible. We found that the YZXJ group had a decrease in the* Firmicutes/Bacteroidetes* ratio, which was identical with those reported in literature. Studies have shown that the ratio of* Firmicutes/Bacteroidetes* is related to fatigue, and the higher the ratio, the severer the fatigue, and vice versa [61–63]. At genus level, the abundance of* Parabacteroides*, unidentified* Saprospiraceae*, and *Elizabethkingia* in* Bacteroidetes* were increased and* Arcobacter, Marinobacter*,* Alkanindiges, Sulfuricurvum, Haliangium, and Thiobacillus* belonging to* Proteobacteria* were decreased in YZXJ group. A large amount of data confirmed that* Bacteroides* can promote the metabolism of ginsenoside that indirectly improves the state of physical fatigue [64, 65].* Proteobacteria* mainly include* Helicobacter* and* Shigella*, both of them can promote the occurrence of CRC by inducing inflammatory reaction and also play an important role in causing CRF [66–68]. According to a research,* Proteobacteria* can affect the deglycosylated metabolism of panax notoginseng saponins via regulating the activities of glycosidases, while upregulation of* Bacteroidetes* may promote the redox metabolism of this drug through improving related enzymes activities in intestine [69].

There is still lack of systematic study in depth on the elevated effects of YZXJ on certain beneficial intestinal bacteria. However, it can be seen that some components of YZXJ have an antimicrobial effect. Oleanolic acid and ursolic acid, the main components of YZXJ, extracted from Fructus ligustri lucidi, can inhibit the transcription of genes related to peptidoglycan biosynthesis, thereby preventing bacterial growth which manifested a strong lethality on* Streptococcus* [70]. Other ingredients, such as Mock strawberry herb, can directly inhibit epithelial-mesenchymal transition (EMT) in tumor cells and can also eliminate pathogenic bacteria like* Streptococcus* [71]. The active ingredient of Scutellaria barbata is essential oil, which has an elimination effect on a variety of bacteria, especially gram-positive bacteria including methicillin-resistant Staphlococcus aureus [72]. Changes in the intestinal microbiota initiate both inflammatory reactions and metabolism disorders, glucose metabolism in especial, which ultimately induce CRF.

This study did not perform metabolomics analysis and in vitro experiments that could not fully elucidate the exact mechanism of YZXJ on alleviating CRF. Nevertheless, this study has clarified that YZXJ was obviously effective in improving physical and mental fatigue in mice with orthotopic colorectal cancer, from which we almost can speculate that this effect is exerted by altering intestinal microbiota, reducing inflammation, and improving metabolism, which hence shows good prospects for application.

## Figures and Tables

**Table 1:** Concentration of IL-1*β*, IL-1*α*, IL-2, IL-6, IL-10, TNF-*α*, and INF-*γ* in NS and YZXJ groups. *∗P*<0.05.

Immune factors	NS	YZXJ	t	*P*
IL-1*β∗*	17.07±7.84	10.29±7.86	2.15	0.04
IL-1*α*	14.34±14.55	18.32±13.33	0.70	0.49
IL-2	12.01±2.07	11.08±1.33	1.29	0.21
IL-6*∗*	229.44±117.54	92.40±71.43	3.40	0.002
IL-10	443.61±227.34	413.29±234.43	0.33	0.75
TNF-*α*	65.56±40.45	69.84±17.07	0.33	0.75
INF-*γ*	193.16±348.26	158.33±250.25	0.28	0.78

**Table 2:** Differential fecal microbiota on genus level between NS and YZXJ groups.

Differential cecal microbiota	Mean		*P*
	NS	YZXJ	
*Increased in the YZXJ group*			
Cellulosilyticum	0	6.83 E-04	0.000999
[Ruminococcus]_gauvreauii_group	2.05E-05	1.85 E-03	0.008991
[Eubacterium]_ventriosum_group	1.05 E-04	7.68 E-04	0.02997
Roseburia	5.46E-05	6.03 E-04	0.032967
Christensenellaceae_R-7_group	2.27E-06	3.35 E-04	0.045954
Terrisporobacter	0	1.06 E-04	0.012987
Candidatus_Soleaferrea	1.59E-05	5.40E-05	0.013986
unidentified_Clostridiales_vadinBB60_group	1.14E-05	6.88E-05	0.02997
Parabacteroides	1.58 E-02	3.58 E-02	0.038961
unidentified_Saprospiraceae	0	1.49E-05	0.010055
Elizabethkingia	0	1.12E-05	0.035984
Microbacterium	0	1.49E-05	0.010055
Alpinimonas	0	1.12E-05	0.035984
Pirellula	3.87E-05	8.74E-05	0.030969

*Decreased in the YZXJ group*			
Faecalibacterium	8.19E-05	0	0.000999
Subdoligranulum	3.41E-05	0	0.000999
Ruminococcaceae_UCG-002	2.05E-05	0	0.000999
Ruminococcus_2	2.73E-05	0	0.000999
Clostridium_sensu_stricto_7	1.36E-05	0	0.008304
Holdemanella	2.05E-05	0	0.000999
Phascolarctobacterium	1.14E-05	0	0.018453
Dialister	5.00E-05	0	0.000999
Tumebacillus	9.10E-06	0	0.041006
Haliangium	1.59E-05	0	0.003737
Thiobacillus	1.36E-05	0	0.008304
Alkanindiges	1.14E-05	0	0.018453
Marinobacter	1.14E-05	0	0.018453
Arcobacter	6.14E-05	0	0.000999
Sulfuricurvum	9.10E-06	0	0.041006
Rubrobacter	1.14E-05	0	0.018453
Iamia	1.82E-05	0	0.001681
Sneathia	9.10E-06	0	0.041006

## Data Availability

The data used to support the findings of this study are available from the corresponding author upon request.
